# The Relationship Between Attitudes Towards Academic Self‐Efficacy and Self and Peer Assessment in Turkish Nursing Students: A Cross‐Sectional Study

**DOI:** 10.1002/nop2.70094

**Published:** 2024-11-21

**Authors:** Olga İncesu, Sevim Ulupınar

**Affiliations:** ^1^ Skill Laboratory, Florence Nightingale Faculty of Nursing Istanbul University‐Cerrahpaşa Istanbul Turkey; ^2^ Department of Nursing Education, Florence Nightingale Faculty of Nursing Istanbul University‐Cerrahpaşa Istanbul Turkey

**Keywords:** academic self‐efficacy, attitude, nursing students, peer assessment, self assessment

## Abstract

**Objectives:**

Academic self‐efficacy (ASE), as well as self (SA) and peer assessment (PA), is associated with the subjective judgements of the individual. The correlation among these concepts merits further research. This study aims to examine the correlation between nursing students' attitudes towards ASE and SA and PA.

**Design:**

This is a correlational cross‐sectional study.

**Methods:**

The sample consisted of 1401 nursing students from five universities in Istanbul, Turkey. The study employed the proportional quota sampling method. The data were collected using a Personal Information Form, View Scale for Peer and Self‐Assessment and ASE Scale. Descriptive statistics and multiple linear regression model (stepwise) tests were used to analyse the data.

**Results:**

Descriptive statistics revealed that 82.2% of the participants were female and their Academic Grade Point Average was 3.00 ± 0.48. As a result of the regression analysis, it was determined that students' age, gender, perception of academic achievement, status of receiving PA training and attitudes towards doing PA and self‐assessment significantly predicted their ASE score.

**Conclusions:**

Students' ASE was found to be medium‐level, their attitudes towards peer and self‐assessment were high and SA and PA attitudes was associated with ASE.

**Patient or Public Contribution:**

A total of 1401 undergraduate nursing students from five undergraduate universities participated in the study and responded to questions on ASE, peer assessment, self assessment, etc.

## 1 Introduction

Nursing education is a practical education involving complex learning. Nursing students go through many learning experiences in settings such as classrooms, clinics, laboratories, communities, etc. during their education. These experiences can either be positive or negative. During this process, students are expected to be able to overcome the problems they face and achieve academic success. Students' problem‐solving skills and academic success are associated with their belief in self‐efficacy (Seçer et al. 2022). Self‐efficacy affects individuals' effort, resistance and stability (Bandura 1982). Individuals with high self‐efficacy appear to have higher personal resilience (Cuartero and Tur 2021; Warshawski 2022) and emotional intelligence (Cuartero and Tur 2021).

Self‐ (SA) and peer assessment (PA), both of which are related to the individual's performance, have a positive effect on the self‐efficacy and self‐regulation skills of students (Panadero, Jonsson, and Botella 2017) and PA increases academic self‐efficacy (ASE) (Fertelli and Tuncay 2020). There are studies in the literature that reveal attitudes and self‐efficacy levels regarding SA and PA. However, there is no study that simultaneously reveals the attitude towards self‐ and PA, ASE level and the relationship between SA and PA attitudes and ASE. Revealing this relationship is important in terms of using SA and PA methods in nursing education and increasing students' ASE. It would be useful for nursing educators to plan initiatives to raise students' perceptions of ASE and to train students for effective SA and PA. The investigation of the factors that affect ASE and SA and PA would contribute to raising the awareness of educators with respect thereto.

## 2 Background

Self‐efficacy is an individual's subjective judgement of his/her performance. Perception of self‐efficacy is related to four sources: (1) Previous success experiences: As the individual becomes successful, his self‐efficacy increases; but unsuccessful performances can also reduce self‐efficacy. (2) Experiences of others: when the individual sees that a model that he thinks is similar to himself, successfully completes a similar task, his belief that he can do it himself increases. For example, seeing a peer successfully complete a performance makes the person feel ‘I can do it too’. (3) Verbal support from others: The positive feedback given to the individual by others (a trainer or peer) during his/her performance positively contributes to his/her self‐efficacy. (4) Physical and emotional state: One of the ways to affect the perception of self‐efficacy is to reduce the individual's stress and to change their negative comments in emotional or physical reactions. The change in the individual's thoughts about how he perceives his performance also affects his self‐efficacy positively (Bouih, Nadif, and Benattabou 2021; Aktay 2021; Bandura 1993).

In nursing education as a practical education, students spend approximately half of their entire education life in the clinical setting, starting from the first year of their education. Factors such as motivation, self‐efficacy and stress affect the clinical success of the student (Zengin et al. 2014). It has been found that students with low self‐efficacy are less likely to be motivated clinically and tend to avoid new learning due to their fear of making mistakes (El‐Sayed, Mousa, and Abd‐Elhamid 2021). A study conducted by Açıksöz, Uzun, and Arslan (2016) with students who had no previous clinical experience showed that as the self‐efficacy levels of nursing students rose, their anxiety towards clinical practice reduced (Açıksöz, Uzun, and Arslan 2016). It is stated that nursing students with low self‐efficacy tend to conduct malpractice more (Baykal and Yildirim 2020), while students with high self‐efficacy have high professional attitudes (Zhou et al. 2022).

ASE ‐one type of self‐efficacy‐is the self‐confidence and belief that an individual can accomplish an academic task (Bulfone et al. 2022; Atılgan and Güngörmüş 2018; Honicke and Broadbent 2016). This refers to the individual's perception of what he/she can achieve with he/she possesses rather than the abilities, knowledge and physical and psychological characteristics he/she possesses (Aktay 2021). The studies have emphasised that ASE is also important for academic achievement and many academic performances (Bulfone et al. 2022; Honicke and Broadbent 2016; Bandura 1993). The students who perceived themselves as academically successful were observed to have higher levels of ASE (Kaplan and Güngörmüş 2022; Özvurmaz and Mandıracıoğlu 2018; Uzdil and Günaydın 2022). It has been observed that students with high ASE cope effectively with academic problems (Ökten and Seferoğlu 2022; Yorulmaz 2019) and students experience more burnout as their ASE drops. (Zhou et al. 2022).

There are a limited number of studies that monitored the long‐term ASE levels of nursing students. The study by Bulfone et al. (2021) monitored the ASE levels of nursing students for 3 years and found that the student's ASE did not significantly change over time (Bulfone et al. 2021). On the other hand, the study by El‐Sayed, Mousa, and Abd‐Elhamid (2021) revealed that the ASE levels of nursing students lowered significantly from the first to the fourth year (El‐Sayed, Mousa, and Abd‐Elhamid 2021). The lack of change or decline in the ASE level of students in long‐term monitoring suggests that students may be in a learning atmosphere where they lack positive experiences or insufficient support for negative experiences. It is important to identify and improve these factors through future studies.

It appears that the correlation between ASE and academic motivation is frequently investigated. The studies by Taheri‐Kharameh et al. (2018), Kaplan and Güngörmüş (2022) revealed that students with high ASE were also more academically motivated (Kaplan and Güngörmüş 2022; Taheri‐Kharameh et al. 2018). Moreover, it was observed that students with high ASE exerted more effort on academic tasks and accomplished these tasks with more planning (Yorulmaz 2019). While Kaplan and Güngörmüş (2022) stated in their studies on the correlation between ASE and academic task procrastination that students with high ASE did not engage in academic task procrastination behaviour, Atılgan and Güngörmüş (2018) put forward that neither did this make a difference (Atılgan and Güngörmüş 2018; Kaplan and Güngörmüş 2022).

ASE is also closely associated with decision‐making mechanisms. In their study, Urgancı and Gürgan (2019) reported that as the ASE levels of young individuals rose, their personal indecisiveness fell and therefore their decision‐making skills increased. The regression analysis showed that personal indecision accounted for 28% of ASE (Urgancı and Gürgan 2019). In their study, Seçer et al. (2022) found that ASE was positively correlated with cautious decision‐making and negatively correlated with avoidant, procrastinatory and panic decision‐making (Seçer et al. 2022).

The use of SA and peer‐assessment – one of the student‐centered assessment‐evaluation methods based on student performance – is gradually increasing. SA – one of the student‐centered assessment‐evaluation methods – is the judgement of the student by assessing his/her works according to specific criteria (Hati, Yunita, and Dewi 2021; Panadero, Jonsson, and Botella 2017). SA allows the student to participate in his/her learning process, recognise his/her weaknesses and strengths, notice his/her learning needs, raise the motivation and improve the self‐confidence of the student who assumes responsibility for learning and develop critical thinking skills (Hati, Yunita, and Dewi 2021; Khonamri et al. 2021; Piper, Morphet, and Bonnamy 2019). SA guides the learner to control and organise his/her performance/studies and enhances the quality of his/her performance/studies (Andrade and Du 2007). PA is the process of assessing the learner's work by peers with similar learning levels within the framework of specific criteria (Fertelli and Tuncay 2020; Tornwall 2018). While doing PA, the student also contributes to his/her learning process (Setiawan, Mardapi, and Andrian 2019). PA encourages deep learning and SA by getting a lot of feedback from different perspectives (Tornwall 2018). PA is also reported to have positive effects on communication, self‐regulation and metacognitive skills and be useful in the development of clinical skills (Tait‐McCutcheon and Knewstubb 2018). Although they have features that influence each other, the literature lacks a study investigating nursing students’ attitudes towards ASE, SA and PA together.

## 3 Methods

### 3.1 Aim

This study aims to examine the correlation between nursing students' attitudes towards ASE and SA and PA assessment.

The study sought answers to the following questions:

1. What are nursing students' levels of ASE?
2. What are nursing students' levels of views on SA and PA?
3. Is there any correlation between nursing students’ perception of ASE and their views on SA and PA?
4. What are the variables affecting perceptions of the ASE of nursing students?

### 3.2 Design

The study was designed as cross‐sectional and correlational.

### 3.3 Setting and Participants

The population of the study consisted of a total of 2940 undergraduate nursing students attending two public and three private universities. It was observed that the sample size should be at least 542 taking into account the confidence interval of 99% and the margin of error of 5%. Using the proportional quota sampling method, stratification was performed as public and private universities and data were collected from each school by using the non‐probability random method. The study reached 1463 students. 20 data collection tools were not included in sampling due to missing data. Among the remaining 1443 data collection tools, 42 including outlier data, were also excluded. A total of 1401 students, including 892 (63.6%) from the public universities and 509 (36.3%) from the private universities, participated in the study (Table 1).

**TABLE 1:** Distribution of the participants.

University	Total students (*N* = 2940)	Ratio	Minimum required students (*n* = 542)	Students included in studies (*N* = 1401)	
				*n*	%
Public	2114	2114/2940 = 0.719	389	892	0.636
Private	826	826/2940 = 0.28	153	509	0.363

### 3.4 Data Collection Tools

The data was collected from students who volunteered to study between 1 November and 31 December 2022. The appropriate time was determined in cooperation with the institutions to collect the data. Informed approval has been obtained prior to participation. They said the respondents' answers to the questionnaire would be confidential. Data were collected from students during extracurricular time allocated for data collection. It took an average of 20 min to fill out the forms. After the participants completed the forms, they handed them over to the researcher. Data were collected through Personal information form, Academic Self‐Efficacy Scale (ASES) and View scale for peer and self assessment (VSfPSA).

#### 3.4.1 Personal Information Form

The form prepared by the researchers consists of a total of 18 questions. The form contains questions about the social‐demographic characteristics of students, such as age, gender, class, school, Academic Grade Point Average (AGPA), and order of choice of school. Questions about the level of perception of the student's academic success and the experience of SA and PA are also available in the form.

#### 3.4.2 Academic Self‐Efficacy Scale

The scale was developed by Jerusalem and Schwarzer (1981) to measure the level of ASE. It is a four‐point Likert‐type scale (4: completely fits me, 1: does not fit me at all) and consists of seven items and one dimension. The minimum and maximum scores of the scale are 7 and 28 points, respectively. The Cronbach's α coefficient was found to be 0.87. Yilmaz, Gürçay, and Ekici (2007) adapted the scale into Turkish and found that Cronbach's *α* coefficient of the scale was 0.79. In the present study, it was calculated that Cronbach's *α* coefficient of the scale was 0.66. Uzdil and Günaydın (2022) and Baykal and Yildirim (2020) where the scale was used, Cronbach's *α* value was 0.66 and 0.70 respectively.

#### 3.4.3 View Scale for Peer and Self Assessment

The scale was developed by Yuen (1998) to measure attitudes towards SA and PA. The scale has a five‐point Likert structure and consists of two sub‐dimensions. The scale includes a total of 13 items. While peer assessment subscale (PAS) consists of six items related to PA, self assessment subscale (SAS) consists of seven items related to SA. The minimum and maximum scores of the scale are 13 and 65 points, respectively. Uysal (2008) adapted the scale to Turkish and found that its Cronbach's *α* coefficient was 0.60 for PA and 0.74 for self assessment. In the present study, the Cronbach's *α* coefficient was 0.79 for the PA dimension, 0.63 for the self assessment and 0.79 for the overall scale. İncesu and Ulupinar (2023) where the scale was used, Cronbach's *α* value was 0.86, 0.60 and 0.83 respectively.

### 3.5 Data Analysis

The IBM SPSS 25.0 software was used to analyse the data. The data appeared to be distributed normally. Minimum, maximum, mean, standard deviation, median, frequency and percentage were utilised to analyse descriptive results. Cronbach's *α* internal consistency coefficient was used to assess the reliability of the scales. The variables predicting ASE and SA and PA scores were analysed using a multiple linear regression model (stepwise method). The categorical variables were included in the model by creating a dummy variable. The data were accepted as significant at the confidence interval of 95% (*p* < 0.05).

## 4 Results

The mean age of the participants was 20.43 ± 1.72 (min = 17, max = 38) years and the AGPA was 3.00 ± 0.48 (min = 1, max = 3.97). 82.2% of the students were female and 30.8% were 2nd‐year students. 86.3% of the students got a score of 2.5 or more points on AGPA and 52.7% of them found their academic achievement at a moderate level. 71.7% stated that they did not participate in any scientific activities related to nursing, 34.3% stated that they would like to work as a nurse and 30.2% as a manager nurse after graduation. It was determined that 86% of the students did not receive any training on SA and 74.5% did not receive any training on PA; whereas, 63.8% had done SA and 30% had done PA during their education. 88.7% of the students stated that they would be objective when doing SA, 88.2% stated that they would be objective doing PA, 56.5% asked for their SA scores and 44.8% asked for their PA scores to be added to their course success grade (Table 2).

**TABLE 2:** The findings related to students' descriptive characteristics, ASE and SA and PA (*n* = 1401).

Variables		*n*	%
Gender	Female	1151	82.2
	Male	250	17.8
University year	1st year	376	26.8
	2nd year	431	30.8
	3rd year	273	19.5
	4th year	321	22.9
AGPA (*n* = 1149)	Below 2.5	157	13.7
	2.5 and above	992	86.3
How do you rate your academic achievement?	Highly successful	59	4.2
	Successful	546	39
	Moderate	739	52.7
	Poor	57	4.1
Do you participate in scientific activities related to nursing?	Yes	397	28.3
	No	1004	71.7
What is your career goal after graduation?	Nurse	481	34.3
	Nurse manager	283	20.2
	Academician	423	30.2
	Self‐employment	138	9.9
	Other	75	5.4
Have you received any training on SA before?	Yes	196	14.0
	No	1205	86
Have you received any training on PA before?		357	25.5
		1044	74.5
Have you done self assessment during your education life?		894	63.8
		507	36.2
Have you done PA during your education life?		421	30.0
		980	70.0
Do you think you can be objective in SA?		1243	88.7
		157	11.2
Do you think you can be objective in PA?		1235	88.2
		165	11.8
Would you like your self assessment score to be added to your course grade?		791	56.5
		609	43.5
Would you like your PA score to be added to your course grade?		628	44.8
		772	55.1

Abbreviation: AGPA, academic grade point average.

Accordingly, their mean scores were 43.23 ± 7.30 in VSfPSA, 24.85 ± 3.75 in SAS, 18.38 ± 4.78 in PAS and 18.53 ± 3.19 in ASES. A low positive correlation was found between ASES and VSfPSA (*r* = 0.222), PAS (*r* = 0.170) and SAS (*r* = 0.217) (Table 3).

**TABLE 3:** Mean scores of ASE, self assessment and PA and their correlation (*n* = 1401).

	Min‐Max	Mean ± SD	1	2	3	4
ASES	9–28	18.53 ± 3.19	**—**	*r* = 0.222*	*r* = 0.170*	*r* = 0.217**
VSfPSA	24–63	43.23 ± 7.30	**—**	1	*r* = 0.890*	*r* = 0.813**
PAS Score	6–30	18.38 ± 4.78	**—**	**—**	1	*r* = 0.458*
SAS Score	15–34	24.85 ± 3.75	**—**	**—**	**—**	1

Abbreviations: ASES, Academic Self‐Efficacy Scale; PAS, Peer Assessment Scale; SAS, Self‐Assessment Score; VSfPSA, View Scale for Peer and Self‐Assessment.

* *r*=Pearson's correlation.

** *p* < 0.01.

Table 4 shows the findings related to the variables that predicted the ASES score. Age, gender, school, university year, AGPA score, perception of current academic achievement, whether or not to receive training on SA and PA and whether or not to do SA and PA, as well as SAS and PAS scores were added to the model as independent variables. The model was found to be statistically significant (*F* = 27.268, *p* < 0.001) and the remaining variables in the model accounted for 15.5% of ASE. It was found that as the age of the students increased, the ASE level would raise by 0.200 times and when females were taken as a reference, the ASE score of males would be 1.181 times higher. When those who perceived their academic achievement as highly successful were taken as reference, it was determined that the ASE score of those who perceived their academic achievement as successful, moderate and poor would gradually decrease (*p* < 0.05). It was found that the self‐efficacy score of those who had not previously received training on PA would decrease −0.534 times compared to those who received training on PA and the self‐efficacy score of those who did not did PA would decrease −0.430 times compared to those who did (*p* < 0.05). It was observed that a one‐unit increase in the SAS score would raise the ASES score 0.161 times (*p* < 0.01).

**TABLE 4:** Variables predicting ASE score.

Model	*B*	SE	Beta	*t*	Sig.	95.0% Confidence Interval for B		Correlations			VIF
						Lower Bound	Upper Bound	Zero‐order	Partial	Part	
(Constant)	12.999	1.340		9.704	< 0.001**	10.371	15.627				
Age	0.200	0.052	0.107	3.883	< 0.001**	0.099	0.302	0.151	0.114	0.105	1.037
Gender (RF: female)											
Gender (male)	1.181	0.227	0.145	5.208	< 0.001**	0.736	1.625	0.136	0.153	0.141	
Academic achievement (RF: highly successful)											
Academic achievement (poor)	−3.545	0.594	−0.231	−5.968	< 0.001**	−4.711	−2.380	−0.096	−0.174	−0.162	2.034
Academic achievement (moderate)	−2.658	0.445	−0.416	−5.971	< 0.001**	−3.531	−1.784	−0.192	−0.174	−0.162	6.588
Academic achievement (successful)	−1.328	0.448	−0.204	−2.962	0.003*	−2.207	−0.448	0.178	−0.087	−0.080	6.404
Have previously received any training on PA (RF: Yes)											
Have previously received any training on PA (No)	−0.534	0.212	−0.073	−2.523	0.012*	−0.950	−0.119	−0.121	−0.075	−0.069	1.141
Have previously done PA (RF: Yes)											
Have previously done PA (No)	−0.430	0.205	−0.061	−2.097	0.036*	−0.831	−0.028	−0.136	−0.062	−0.057	1.163
Self assessment score	0.161	0.023	0.191	6.969	< 0.001**	0.115	0.206	0.218	0.202	0.189	1.022

*Note:* Multiple Linear Regression, Dependent Variable: Academic Self‐Efficacy Total Score, RF, VIF. Model *F* = 27.268, *p* < 0.001, Adj *R* ^2^ = 0.155, SE of the Estimate = 2.93479, Durbin Watson = 1.883.

Abbreviations: RF, reference; VIF, variance inflation factor.

* *p* < 0.05.

** *p* < 0.01.

## 5 Discussion

To our best knowledge, this is the first study to attempt to show the correlation of SA and PA with ASE in the same study. In the current study, we investigated the relationship between ASE and SA and PA among Turkish undergraduate nursing students.

The ASE of the students was found to be moderate. The results of studies on nursing students using the same scale (Fertelli and Tuncay 2020; Ülgen and Köksoy 2022) are similar to the results of the present study. There are also studies that reported lower (Uzdil and Günaydın 2022; Baykal and Yildirim 2020) and higher (Küçükkaya, Özdemir, and Süt 2022; El‐Sayed, Mousa, and Abd‐Elhamid 2021; Zhou et al. 2022; Rohmani and Andriani 2021) ASE scores of students. It is recommended that initiatives be undertaken to improve the student's ASE. Regular feedback from educators to students, peer‐assisted learning, peer mentoring and more frequent use of PA in the curriculum may be helpful. Furthermore, increased use of collaborative learning methods in curricula can contribute to ASE by increasing peer interaction. Monitoring a student's own development through portfolios and skill report cards can contribute to increasing ASE. Long‐term monitoring of student ASE levels will also be useful for early detection of changes and planning initiatives to do so.

The scores of the students on the SA and PA scales were also above average. It was observed that most of the students did not receive training on SA and PA; however, more than half of them stated that they did SA and one‐third of them did PA during their educational lives. Although the rate of students who considered that they would be objective while doing SA and PA was quite high, the rate of those who asked for SA and PA scores to be added to the course success grade was remarkably low. Based on these results, it can be asserted concluded that the students had positive attitudes towards SA and PA, but they were concerned that they may not be objective and professional when assessing since they were not trained in this subject. In the studies of Kaufman and Schunn (2011) and Yang (2019), students stated that they did not consider their peers as qualified to evaluate their performance and the feedback of their peers was of poor quality. Increased use of rubrics and checklists may help reduce students' anxiety about being objective. In addition, rubrics and checklists can support learning by improving the performance of students (Jelovsek et al. 2013). It is also recommended that training be conducted on this subject in order to be able to do SA and PA in a more qualitative way.

A positive correlation was detected between ASE and SA and PA attitudes.In the study, it was observed that age, gender, the perception of academic achievement, status of receiving training on PA, doing PA and having a positive attitude towards SA affected the perception of ASE. The fact that ASE positively increased with increasing age can be associated with the increase in the student's experiences. The study by Cuartero and Tur (2021) reported that there was no significant difference between age groups in terms of ASES scores; whereas, the study by Özgül and Diker (2017) reported that age was an effective variable. The study found that men had higher academic self‐efficacy. While similar results were obtained in the studies by Kaplan and Güngörmüş (2022) and Özvurmaz and Mandıracıoğlu (2018). On the other hand, some studies show that women have higher ASE (Ülgen and Köksoy 2022; Küçükkaya, Özdemir, and Süt 2022; Seki Öz et al. 2021; Warshawski 2022; Sachitra and Bandara 2017; Bouih, Nadif, and Benattabou 2021) and some studies also show that ASE does not change according to gender (Cuartero and Tur 2021; Atılgan and Güngörmüş 2018; Özgül and Diker 2017; Basith, Syahputra, and Ichwanto 2020; Baykal and Yildirim 2020). Based on the literature, it is not possible to draw a definite conclusion about the correlation between gender and ASE. But it may be useful for educators, especially in countries where paternalistic culture is active, to take into account that girls may have a low level of ASE.

Contrary to widespread belief in the literature, the study found no relationship between AGPA and ASE. The literature includes studies that report a positive correlation between academic achievement and self‐efficacy (Bouih, Nadif, and Benattabou 2021; Bandura 1993) and ASE (Al Sebaee, Aziz, and Mohamed 2017; Bulfone et al. 2022; Basith, Syahputra, and Ichwanto 2020; Honicke and Broadbent 2016), there are also studies indicating a low and negative correlation (Küçükkaya, Özdemir, and Süt 2022). The meta‐analysis study by Richardson, Abraham, and Bond (2012) indicated that, belief in ASE was as effective as 9% on the academic achievement mean score. It is also a finding worthy of further research that the AGPA of the students was not effective on their perception of achievement. It should be analysed whether this result is due to the student's inability to do an objective self assessment or his/her attitude towards the assessment and evaluation system.

Although there was no correlation between AGPA and ASE in the study, it was observed that the perception of academic achievement was effective and the ASE score also increased as the student felt successful. Similarly, studies have shown that students who perceive themselves to be academically successful have higher ASES scores (Kaplan and Güngörmüş 2022; Uzdil and Günaydın 2022; Özvurmaz and Mandıracıoğlu 2018). This shows that the perception of self‐efficacy is subjective, but it is consistent with the nature of it. However, under these circumstances, it should be taken into account that a successful student may consider himself academically inadequate or that a failed student may perceive himself as academically adequate. The study by Kumandaş and Kutlu (2013) also reported that while students with high academic achievement perceived their performance as lower, students with poor academic achievement perceived their performance as better than it was. In line with this finding, we think it would be useful for educators to ask students to make an immediate SA of their performance and to give them realistic and regular feedback.

Although receiving training on PA and doing PA were among the variables affecting ASE in the study, PA attitude score was not found to be correlated. This finding was a significant result in terms of performing the behaviour and having knowledge being more effective on self‐efficacy perception than the individual's attitude. It indicates that students should be encouraged to take action and perform the behaviour. PA has effects such as recognising students' weaknesses and strengths and increasing their critical thinking skills (Akpınar and Kranda 2016), raising self‐confidence (Altıntaş and Karaaslan 2019) and contributing to professional development (Bergum et al. 2017). On the other hand, it is known that difficulties such as anxiety about getting and giving negative feedback, anxiety about being criticised for one's personality, fear of conflict and unwillingness to assume responsibility for PA (Akpınar and Kranda 2016; Takase, Yamamoto, and Sato 2018) are also experienced. Therefore, it is considered that training on PA, increased PA and utilising objective tools such as rubrics and checklists during this assessment would contribute to students' ASE by alleviating their concerns about being objective.

It was remarkable that while there was no correlation between having been trained on SA and doing SA and ASE, SA attitude positively affected the perception of ASE. The meta‐analysis study by Panadero, Jonsson, and Botella (2017) reported that SA had positive effects on students' self‐efficacy and ability to organise their learning and the meta‐analysis study by Sitzmann et al. (2010) demonstrated that there was a moderate correlation between self‐efficacy and SA. Based on these results, it can be asserted that while attitude is more determinative in SA, behaviour is more determinative in PA.

### 5.1 Limitations and Strengths

The limitations of the study can be summarised as follows: First, the data were collected a single time in the study using a cross‐sectional research design. Moreover, all data collection tools were answered with students' self‐report. Responses involving attitude may change over time, which is considered a limitation.

Despite the stated limitations, this study has several strengths. This study is the first attempt to show the correlation of SA and PA with ASE. The sample size of the study is quite large. Finally, regression analysis results can guide interventions to increase positive attitudes towards ASE and self‐ PA.

## 6 Conclusion

This study showed that the level of ASE of nursing students still needs to be improved and that students have positive attitudes towards self‐ and peer‐assessment. It also revealed that SA and PA contributed to the development of ASE. In the study, it was also emphasised that students need more experience with the PA method. In addition, some demographic characteristics of the students as well as the perception of academic achievement as opposed to academic achievement score were found to be effective in the development of ASE.

## 7 Implications and Recommendations

In nursing education, various interventions can be planned to increase the level of ASE of nursing students. Since SA and PA are methods that support each other (İncesu and Ulupinar 2023), using them together in learning environments may contribute to increasing the level of ASE. In addition, since feedback given by the educator increases ASE (İncesu and Şahin Bayındır 2024), it may be useful to give regular feedback to nursing students by the educator in theoretical and clinical practice. Since the PA method significantly increases the students’ ASE level (Fertelli and Tuncay 2020), it may contribute to increase the ASE levels of students to receive training on how to perform the PA method and to have more PA experiences. Considering the relationship between ASE and the perception of academic success, monitoring the student's own development with portfolios, skill report cards, etc. can be effective in forming his/her own subjective judgement.

## Author Contributions

O.İ., S.U.: study design, critical revisions for important intellectual content. O.İ.: data collection, data analysis, drafting of manuscript.

## Ethics Statement

Istanbul University‐Cerrahpaşa Social and Human Sciences Ethics Committee (Date: 04/10/2022, Decision number: 2022/312).

## Conflicts of Interest

The authors declare no conflicts of interest.

## Data Availability

The data that support the findings of this study are available from the corresponding author upon reasonable request.
